# New Technologies for the Management and Rehabilitation of Chronic Diseases and Conditions

**DOI:** 10.1155/2015/180436

**Published:** 2015-06-24

**Authors:** Gianluca Castelnuovo, Giancarlo Mauri, Susan Simpson, Angela Colantonio, Stephen Goss

**Affiliations:** ^1^Istituto Auxologico Italiano IRCCS, Psychology Research Laboratory, Ospedale San Giuseppe, 28824 Verbania, Italy; ^2^Department of Psychology, Catholic University of Milan, 20123 Milan, Italy; ^3^Department of Informatics, Systems and Communication, University of Milano-Bicocca, 20126 Milan, Italy; ^4^School of Psychology, Social Work & Social Policy, University of South Australia, Adelaide, SA 5001, Australia; ^5^Toronto Rehabilitation Institute, University Health Network, Toronto, ON, Canada M5G 2A2; ^6^Department of Occupational Science & Occupational Therapy, University of Toronto, Toronto, ON, Canada M5G 1V7; ^7^Metanoia Institute, Middlesex University, London W5 2QB, UK

The economic burden of chronic diseases and conditions (such as cardiovascular pathologies, diabetes, obesity, chronic obstructive pulmonary disease, chronic pain, and traumatic brain injuries) requires new solutions not only in traditional clinical settings (in-patient treatments), but also in innovative healthcare scenarios (out-patient long-term monitoring).

New technologies can provide clinicians and patients with many solutions at different levels: diagnostic and monitoring, early risk detection, treatment and rehabilitation, provision of feedback and alerts, and motivational strategies that facilitate changes in dysfunctional behaviors or maintenance of healthy lifestyles. A health technology assessment approach is necessary in order to collect evidence to evaluate the clinical and cost effectiveness of new tools and to strengthen the political choice to use health technologies in clinical fields.

Telemedicine, e-health, and m-health scenarios can improve health outcomes, quality of life, and well-being and facilitate functional patient empowerment and engagement. Mobile technologies in particular can offer advantageous solutions: m-health could be considered an evolution of e-health and defined as the practice of medicine and public health as supported by mobile communication devices. Indeed, the m-health approach has the potential to overcome many of the limitations associated with the traditional, restricted, and highly expensive in-patient treatment of many chronic pathologies.

A range of electronic health systems have been implemented in chronic disease management using stationary and mobile computers, smartphones, and other mobile platforms with differing access to data technology. New innovations in technology are required to meet the challenges associated with overcoming various barriers such as organizational and technological difficulties, lack of technology acceptance, costs of system implementation and maintenance, lack of system interoperability with other informatic tools, reduced communication between clinicians and patients, and difficulties in data processing due to the limitations of devices used in patient monitoring.

There is potential for the development of a new and innovative model of healthcare as represented by such technologies as telemedicine, e-health applications, biomedical sensors and devices, integrated platforms and technologies for remote monitoring and management, web and Internet based clinical protocols, and m-health solutions. This model has the potential to offer healthcare that is tailored to the specific needs of the individual, whilst providing the benefits of a mobile, noninvasive, balanced, integrated, and lifestyle friendly framework that is useful from both a preventative and intervention-focused perspective.

The major aim of this special issue is to bring to light new international developments in the management and rehabilitation of a range of chronic diseases and clinical conditions, such as stroke, dementia, cardiovascular diseases, dialysis patients, or those with facial paresis. A range of technological developments, lifestyle, and environmental factors have been described in relation to these diseases, including a rehabilitation system for stroke patients using vibrotactile feedback, a new system of facial movement analysis for facial paresis, an exploration of the effects of exercise for dialysis and cancer patients, and the influence of environment on those with dementia and coronary artery disease.

In conclusion, as demand for the management of a range of chronic conditions increases in the imminent future, there is likely to be considerable scope for the integration of clinical psychology and medicine. A key future challenge for those working in both traditional and m-health/e-health settings will be to further develop the evidence base for chronic care management in order to enhance technological standards and fine-tune both clinical protocols and organisational models.

*Gianluca Castelnuovo* *Giancarlo Mauri* *Susan Simpson* *Angela Colantonio* *Stephen Goss*

